# Outbreak of listeriosis associated with consumption of deli meats in a hospital, Germany, February to March 2023

**DOI:** 10.2807/1560-7917.ES.2025.30.7.2400316

**Published:** 2025-02-20

**Authors:** Lena Feige, Nicole Walter, Ahmad Fawzy, Tilman Schultze, Melanie Hassel, Manfred Vogt, Philipp Zanger, Anja Schoeps

**Affiliations:** 1 Federal State Agency for Consumer & Health Protection Rhineland-Palatinate, Koblenz, Germany; 2 District Public Health Authority, Ludwigshafen, Germany; 3 Cairo University, Faculty of Veterinary Medicine, Department of Medicine and Infectious Diseases, Cairo, Egypt; 4 Landesbetrieb Hessisches Landeslabor (LHL), Gießen, Germany; 5 Heidelberg Institute of Global Health, University Hospitals, Heidelberg, Germany

**Keywords:** Listeria monocytogenes, Listeriosis, Immunocompromised host, Glucocorticoids, Deli meats, Food safety, Nosocomial infections

## Abstract

*Listeria monocytogenes* can cause severe illness in individuals with weakened immune systems. In March 2023, *L. monocytogenes* was isolated from blood (n = 2) or pleural fluid (n = 1) of three febrile patients receiving synthetic glucocorticoids in a tertiary hospital in Germany. Food supply records suggested sliced parboiled sausage as the likely source, and *L. monocytogenes* was isolated from four samples of sealed packaged sliced sausages and ham from one manufacturer. The patient and food isolates clustered within 0–4 allelic differences. Counts of *L. monocytogenes* in all four food samples were < 100 colony-forming units (CFU)/g, a threshold in the European Union legislation for ready-to-eat products with specific conditions. Our findings, aligned with previous evidence, highlight that persons with weakened immune systems should not be exposed to *L. monocytogenes* in food. We advocate for a clear communication of deli meats as high-risk foods, so individuals with weakened immune systems can adjust their diet to reduce their risk for invasive listeriosis. We recommend an update of dietary and hygiene guidelines for care settings and private homes where food is prepared, handled and stored for persons with weakened immune systems.

**Key public health message**

**What did you want to address in this study and why?**

*Listeria monocytogenes,* a bacterium commonly found in the environment, can cause a severe disease, called invasive listeriosis, particularly in individuals with weakened immune systems. This severe disease can be fatal. Infection typically occurs through the consumption of contaminated food. We aimed to identify the source of invasive listeriosis in a hospital outbreak involving three patients.

**What have we learnt from this study?**

All three patients had eaten sliced poultry mortadella at the hospital on the same day. *Listeria monocytogenes* was found in four samples of unopened packages of sausages and ham from the same manufacturer. Whole genome sequencing confirmed that the packaged sliced sausages were the source of the infection of all three patients.

**What are the implications of your findings for public health?**

Sliced sausages and other cold cuts, even when fully cooked, should be treated as high-risk foods. Persons with weakened immune systems should either avoid these foods entirely or eat them only when freshly sliced. Our findings underscore the need to provide clear dietary and hygiene guidelines for kitchens in care settings and private homes where food is prepared, handled and stored for persons with weakened immune systems.

## Background

*Listeria monocytogenes* is a ubiquitous Gram-positive bacterium commonly found in soil and decaying vegetation. It is mostly transmitted to humans via ingestion of contaminated food and poses a particular risk for humans once disseminated in food-processing environments. *Listeria monocytogenes* tends to persist due to its ability to build biofilms, survive in anaerobic environments, grow at refrigerator temperatures and to withstand elevated levels of salt [1,2]. Taken together, these characteristics make it a challenge to eliminate *L. monocytogenes* from the food production process, even when rigorous hygiene measures are in place.

*Listeria monocytogenes* can multiply in foods with long shelf life, which are stored over days or weeks, even at refrigerator temperatures. These products include soft cheeses, smoked fish and deli meats [3-6]. Accordingly, deli meats, especially sliced and packaged parboiled sausages, Frankfurters and cooked ham have caused multiple invasive listeriosis outbreaks in the past [7-14]. Even though these meats are cooked to a core temperature of > 72°C during production, and thus expected to be free from viable pathogenic bacteria, they can become contaminated during slicing or packaging and subsequently provide good growth conditions for *L. monocytogenes.*

Although exposure to *L. monocytogenes* is likely to be ubiquitous, invasive listeriosis is rare in the overall population with < 1 notified case per 100,000 population per year in the European Union (EU) [15]. While infection with *L. monocytogenes* usually presents with no or only mild symptoms such as influenza-like symptoms or diarrhoea, infection with *L. monocytogenes* during pregnancy may lead to miscarriage, stillbirth, premature birth and neonatal infection. In people with weakened immune systems, infection can lead to invasive listeriosis, which may present as septicaemia or encephalitis and lead to death [16,17]. Case fatality rate of 9–30% for invasive listeriosis is higher than for most other bacterial gastrointestinal infections [18,19]. The infection risk is pronounced in patients with malignancy and in individuals with medication, diagnoses and conditions characterised by impaired T-cell function such as pregnancy [20], increasing age, intake of synthetic oral glucocorticoids [21], anti-TNFα-therapy [22] and AIDS [23]. More specifically, the common determinant for an elevated risk within these patient groups is a weakened antigen-specific CD8+ effector T-cell response, which is thought to be key in eliminating intracellularly replicating listeria, thereby contributing to resistance against and clearance of infection [24].

To mitigate the risk of infections with *L. monocytogenes*, the European Commission (EC) has established microbiological criteria (2073/2005) which regulate *L. monocytogenes* in ready-to-eat (RTE) food products [25]. In foods which support the growth of *L. monocytogenes*, (i) the bacterium should not exceed the limit of 100 colony forming units (CFU)/g during the shelf life of the product. If the manufacturer is not able to demonstrate that the food safety criteria are followed, (ii) *L. monocytogenes* is not to be detected in 25 g sample when the product leaves the production facilities [25]. When RTE foods are intended for infants and for special medical purposes; (iii) *L. monocytogenes* is not to be detected in 25 g sample throughout the shelf life of the product. A recent amendment (2024/2895) to the regulation has extended the second criterion from the time the product leaves the production facility until the end of the shelf life (as criterion (iii)) and included persons with weakened immune systems in the third criterion [26]. In some countries elsewhere, such as the United States (US), *L. monocytogenes* is not to be detected in 25 g of RTE foods in general [26,27].

## Outbreak detection

In March 2023, two cases of invasive listeriosis, with disease onset dates on 4 March and 11 March, were diagnosed in a tertiary care hospital in Rhineland-Palatinate, Germany (Hospital A). The hospital laboratory notified the cases to the local health authority which further informed the Rhineland-Palatinate federal state health authority of their occurrence.

Here, we report a nosocomial point-source outbreak of invasive listeriosis in a tertiary care hospital in Germany (February–March 2023) and the results of the subsequent investigation to identify the source of the infection and prevent further cases.

## Methods

### Surveillance of *Listeria monocytogenes*

Invasive listeriosis is a notifiable disease in Germany, meaning that laboratories must report to the competent public health authorities all cases with finding of *L. monocytogenes* by conventional culture or PCR in normally sterile body fluids. Local public health authorities try to contact the cases or their closest relatives, when the cases themselves cannot be interviewed, to investigate potential sources of the infection, including consumption of foods that are frequently associated with *L. monocytogenes* infections. If a likely source of infection can be identified, the local food control authority is informed about the potentially contaminated food source and usually initiates food sampling at the site where the food was consumed and/or purchased by the patient. If *L. monocytogenes* is detected in these food samples, the local food control authority visits the production site to assess adherence to hygiene measures and to take food and environmental samples at the production site.

### Epidemiological investigation

With the aim to confirm the outbreak, identify further cases and ultimately eliminate the source, we initiated an epidemiological investigation. We created an outbreak team consisting of representatives of the departments of microbiology and hygiene of Hospital A and the local and regional public health authorities. We then established a case definition that was used to screen the laboratory information system of Hospital A.

A probable case of invasive listeriosis was defined as a patient with samples from normally sterile fluids tested positive for *L. monocytogenes,* samples analysed about 1 month prior and after the index case which was diagnosed on 5 March. Based hereon, we screened on average 130 cultures per week obtained from fluids, such as blood or pleural fluid, from patients, from 1 February to 31 March and identified a third probable case of invasive listeriosis, with *L. monocytogenes* isolated from pleural fluid on 6 March.

### Collection of food and environmental samples

On 14 March, the food control authority took samples from two unopened packages of mixed sliced sausages (retention samples) and three packages of different unsliced or whole sausages (parboiled turkey and pork sausages and cooked turkey ham) in the kitchen of Hospital A. On 23 March, we conducted an unannounced visit to Meat supplier A, a regional producer, and took altogether 18 surface samples from a cutting board, a scale, sewers, slicers, worktables and washbasins and samples from a parboiled whole turkey sausage and a whole pork ham. On 24 March, the food control authority took six new samples from unopened packaged deli meats in the kitchen of Hospital A. On 26 March, the food control authority visited Caterer A, supplied by Meat supplier A, and took a sample from an unopened packaged sliced cooked pork ham. Meat supplier A was visited again on 3 April and 11 new surface samples were taken from a cold room, cutting boards, a filling machine, sewers, slicers and worktables and samples from seven whole parboiled pork sausages. More details of the samples can be seen in Supplementary Table 1.

All samples were analysed for detection and enumeration of *L. monocytogenes* according to DIN/EN/ISO 11290–1:2017 and 11290–2:2017 at accredited laboratories.

### Whole genome sequencing

Extraction of DNA and library preparation was performed according to the manufacturers’ protocols at federal state public health laboratories, Rhineland-Palatinate, using the Promega Maxwell RSC DNA Extraction Kit (Promega, Madison, US) and the Nextera XT DNA Library Prep Kit (Illumina, San Diego, US). Libraries were sequenced on Illumina MiniSeq in 2 × 150 bp paired-end mode.

Reads were trimmed and filtered using Trimmomatic with a quality Phred score threshold of 30 on a minimum length of 20 nt. De novo assembly was performed using Velvet (https://github.com/dzerbino/velvet) and assembled sequences were mapped to the *L. monocytogenes* EGD-e seed genome (NC_003210.1). Ridom SeqSphere software version 8.5.1 (Ridom GmbH, Münster, Germany) was used for trimming of raw reads, genome assembly, assessment of differences in alleles and single nucleotide polymorphisms (SNP) using minimum spanning trees (MST), as well as assignment of in silico 5-plex PCR serogroups, seven-locus multilocus sequence type (MLST) and core-genome MLST (cgMLST) complex types (CT) [28]. Core-genome MLST CTs were delineated from assembled sequences covering ≥ 98.8% of 1,701 cgMLST targets, using a threshold of ≤ 10 alleles, and their profiles stored on the cgMLST.org nomenclature server (https://www.cgmlst.org/ncs). Minimum spanning trees were constructed by pairwise clustering that ignores missing values and a threshold of differences of ≤ 7 alleles. The cluster naming was in accordance with the internal nomenclature of the Robert Koch Institute (RKI), Berlin, Germany.

The following web-based tools from the Center for Genomic Epidemiology (https://www.genomicepidemiology.org/) were used: CSI Phylogeny version 1.4 (https://cge.food.dtu.dk/services/CSIPhylogeny/) was used for SNP analysis, ResFinder version 4.1 (http://genepi.food.dtu.dk/resfinder) to determine resistance genes and VirulenceFinder version 2.0 (https://cge.food.dtu.dk/services/VirulenceFinder/) to identify virulence genes. The Interactive Tree Of Life (https://itol.embl.de/) version 5 was used for the visualisation of the SNP-based phylogenetic tree.

Publicly available sequencing data of *L. monocytogenes* MLST sequence type (ST)2 isolates were downloaded in June 2023 from the cgMLST.org server and imported in Ridom SeqSphere. The aforementioned bioinformatical pipeline was used to perform the de novo assembly and mapping of reads to the *L. monocytogenes* EGD-e seed genome (NC_003210.1). Neighbour-joining tree analysis was performed using Ridom SeqSphere to compare the *L. monocytogenes* ST2 sequencing dataset with the outbreak isolate.

## Results

### Epidemiological investigation

**Figure 1:**
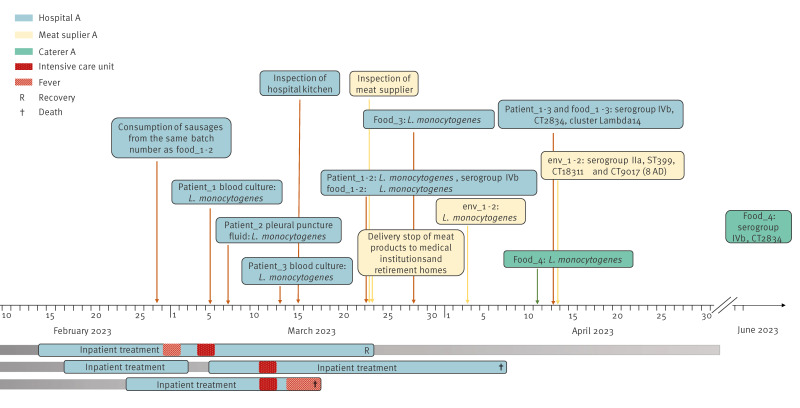
Timeline of an outbreak of *Listeria monocytogenes* in a hospital*,* Germany, 2023 (n = 3)

CT: complex type; Env: environmental isolate; *L:* *Listeria*; ST: sequence type.

In February–March 2023, three cases with *L. monocytogenes* were identified in Hospital A (Figure 1). All three patients developed septicaemia, and *L. monocytogenes* was isolated from blood (n = 2) or pleural fluid (n = 1) samples. The ages of the patients ranged from their fifties to their eighties. The cases had several underlying conditions such as melanoma, heart failure, rheumatoid arthritis and recurrent pericardial effusion. All three patients were treated with synthetic oral glucocorticoids (dexamethasone 4 mg/d, prednisolone 30 mg/d or 40 mg/d). One patient recovered, but the other two patients died.

The dietary history of the cases suggested a common source, and all three had consumed poultry mortadella (Geflügelmortadella) during breakfast or dinner on 27 February (Figure 1). In total, 670 patients of Hospital A had been served packaged sliced parboiled sausage (Wurstaufschnitt) during either breakfast or dinner on 27 February.

No violations in food handling and serving could be identified when the hospital was inspected by the food control authority.

### Food and environmental samples

A total of fifty samples were collected: 11 deli meat samples from Hospital A, 29 surface samples and 9 deli meat samples from Meat supplier A and one deli meat sample from Caterer A. Caterer A and Hospital A were both supplied by Meat supplier A. *Listeria monocytogenes* was detected in four unopened food samples: three packages of mixed sliced parboiled sausages (Wurstaufschnitt) from Hospital A and cooked ham (Hinterkochschinken) from Caterer A (Table). In enumeration, the counts of *L. monocytogenes* in the tested food samples were < 100 CFU/g. Detailed information about the samples is presented in Supplementary Table 1. At Meat supplier A, adherence to hygiene measures was found to be unsatisfactory, primarily due to a shortage of staff, and *L. monocytogenes* was detected in environmental samples from a sewer and a washbasin.

**Table:** Characteristics of *Listeria monocytogenes* isolates from food and environment in an investigation of a listeriosis outbreak in a hospital, Germany, February–March 2023 (n = 6)

ID	Sample type	Sample details	Sampling date	Use-by date^a^	cgMLST	MST cluster	Sampling site
**Food_1**	Food	Mixed packaged sliced sausage (Wurstaufschnitt)	14 Mar	NA	CT2834	Lambda14	Hospital A^b^
**Food_2**	Food	Mixed packaged sliced sausage (Wurstaufschnitt)	14 Mar	NA	CT2834	Lambda14	Hospital A^b^
**Env_1**	Environment	Swab sample from a washbasin	23 Mar	NP	CT18311	Other	Meat supplier A
**Env_2**	Environment	Swab sample from a sewer	23 Mar	NP	CT9017	Other	Meat supplier A
**Food_3**	Food	Packaged sliced parboiled sausage (Bierwurst)	24 Mar	28 Mar 2023	CT2834	Lambda14	Hospital A^b^
**Food_4**	Food	Packaged sliced cooked ham (Hinterkochschinken)	29 Mar	26 Mar 2023	CT2834	Lambda14	Caterer A^b^

cgMLST: core genome multilocus sequence typing; CT: complex type; Env: environment; ID: identification code; MST: minimum spanning tree; NA: not available; NP: not applicable.

^a^ Use-by dates for packaged sliced sausages are generally 8 days after production.

^b^ Deli meats supplied by Meat supplier A.

### Whole genome sequencing

**Figure 2:**
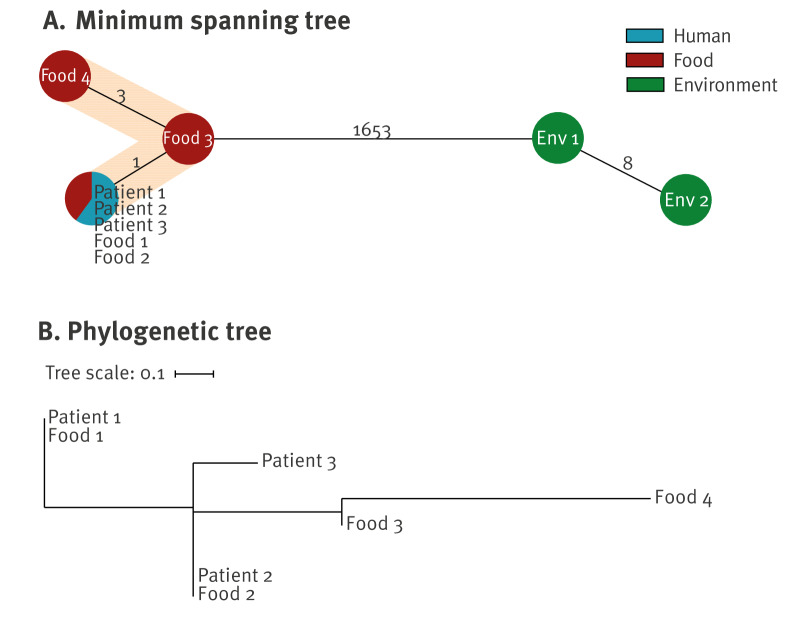
Minimum spanning tree (MST) and single nucleotide polymorphism (SNP) analysis of *Listeria monocytogenes* isolates from patients (n = 3), food (n = 4) and environment (n = 2) in a listeriosis outbreak in a hospital, Germany, February–March 2023

The three patient and four food isolates were closely related. The isolates were of molecular serogroup IV, ST2 and belonged to the same cgMLST CT2834, with differences of 0–4 alleles, forming the cluster Lambda14 (Figure 2, Table). The two environmental isolates belonged to two different CTs within ST399 (CT18311 and CT9017) (Figure 2). The difference between the environmental isolates and the patient and food isolates was > 1,650 alleles. The SNP analysis of the patient and food isolates confirmed these results, with seven SNP differences between the isolates. Isolates of Food_1 and Patient_1 were closest to each other, as well as Food_2 and Patient_2, suggesting potential food-to-human transmission via respective contaminated food products (Figure 2B). Sequencing coverage of all isolates was > 60 × (median 85 ×).

Env: environment.

Core genome multilocus sequence types were delineated from cgmlst.org (https://cgmlst.org/ncs) [28].

Panel A. Minimum spanning tree (MST) comparing alleles of 1,673 loci of the core genome of *Listeria monocytogenes* isolates (n = 9). Isolates are coloured according to their origin. Clusters are defined with a cluster distance threshold of seven allelic differences. Orange background highlights the cluster Lambda14 (sequence type (ST) 2). Numbers present allele differences between nodes of the tree.

Panel B. Phylogenetic tree based on SNP analysis of *Listeria monocytogenes* isolates (n = 7) belonging to cluster Lambda14 rooted on Food_1. Tree scale in substitutions per site.

## Outbreak control measures

On 23 March, when we received the results of detection of *L. monocytogenes* in a sample of packaged sliced mixed sausage (Wurstaufschnitt) from the kitchen of Hospital A, the hospital immediately stopped further deliveries from Meat supplier A (Figure 1). The remaining products from Meat supplier A in the fridge of hospital A were discarded. Hospital A contracted a new supplier for meat products which guaranteed to deliver meat products in which *L. monocytogenes* cannot be detected in 25 g before the use-by date. Moreover, Meat supplier A ceased the distribution of its products to other hospitals and retirement homes. Meat supplier A had to clean and disinfect the complete production site and was closely monitored by the food control authority. No new cases were identified in Hospital A after the change of the meat supplier.

To identify a potential expansion of the outbreak, we compared genomic sequences from publicly available databases and the German national Consultant laboratory (CL) for *Listeria* at the RKI with the outbreak isolates [29]. We did not find any other isolates that clustered with the outbreak isolate. The most closely related isolate at the CL (22 alleles and 79 SNP differences to the isolate Food_1) was a clinical *L. monocytogenes* isolate submitted in 2017 [30]. All the other published sequences were more distant, as can be seen in Supplementary Figure 2.

## Discussion

We could show that packaged sliced deli meat was the likely source of invasive listeriosis in three temporally clustered cases of patients receiving high-dose oral glucocorticoids in a tertiary care hospital in Germany. There could have been more cases than these three, since not all clinical isolates of *L. monocytogenes* obtained from German healthcare settings are sequenced, even though sequencing of such isolates is free of charge.

We did not find *L. monocytogenes* in the sausage or ham samples taken at Meat supplier A but isolated the bacterium from packaged sliced sausage and ham samples from Hospital A. As *L. monocytogenes* was found in samples from unopened packages of different types of sliced sausage and ham, the food was most probably contaminated during production, possibly during slicing or packaging. The packaging process was considered as a possible source of contamination in a recent outbreak in Finland, where consumption of a plant-based product was linked to a nosocomial listeriosis outbreak [31].

Sliced parboiled sausages are among the most frequently consumed foods in Germany and have caused large outbreaks of *L. monocytogenes*, both in nosocomial settings and in the community [9,12]. In 2003, the US Food and Drug Administration (FDA) identified deli meats as the food category with the highest risk of *L. monocytogenes* contamination of RTE foods [4]. In a study from 2022, > 90% of all invasive listeriosis cases in the US were attributable to deli meats [5]. Several other studies have also attributed most listeriosis cases to deli meats [5,6,32]. Despite this high infection risk, deli meats, like sliced parboiled sausages or cooked ham, are not listed as food items that should be avoided by those at an increased risk of invasive listeriosis in the EU [33-35]. In the US and the United Kingdom (UK), deli meats are included in the food categories with the highest risk of infection with *L. monocytogenes* [36,37]. The current German dietary recommendations for individuals with weakened immune systems discourage from consumption of raw or smoked meat and fish, other RTE foods such as various types of cheeses (including cheese preparations from pasteurised milk) and pre-cut and packaged salads but not deli meats [33,34,38,39].

Although we isolated *L. monocytogenes* from three samples of sausage and one sample of ham, the counts of *L. monocytogenes* in these samples were < 100 CFU/g, which was the allowed limit during the shelf life of RTE food products in the EC regulation (2073/2005) [25]. As the sausage, which the patients consumed on 27 February, was not available for investigation, we could not assess the amount of *L. monocytogenes* in this specific sausage. Thus, we do not know if the food product was in compliance with the regulations.

An interruption in the cold chain in the hospital could have led to faster multiplication of *L. monocytogenes*. However, no violations in food handling and serving could be identified when the hospital was inspected by the food control authority.

Despite a dose-response relationship in the risk of invasive listeriosis, persons with weakened immune systems have a higher risk of infection even with lower levels of *L. monocytogenes* contamination [32,40]. Additionally, the increase of the risk of invasive listeriosis for patients with multiple risk factors is unknown, and different strains of *L. monocytogenes* exhibit variations in virulence [40].

The risk of infection could be reduced if other measures were applied such as the non-detectability of *L. monocytogenes* in 25 g of the product before the end of its shelf life (zero tolerance policy), a regulation which exists in the US for all foods [4] and in the EU for foods intended for infants or specific medical purposes, which includes consumers with weakened immune systems in the current amendment (Commission Regulation (EC) No 2073/2005, amendment 2024/2895) [26] but was not in place when the outbreak occurred. Competent authorities need to be vigilant and control that manufacturers follow the regulations, and institutions with vulnerable populations have to make sure that the food they serve has been manufactured according to their requirements.

## Conclusions

Genetically matching *L. monocytogenes* were found in three isolates from patients with invasive listeriosis and in four samples from packaged sliced parboiled sausages and cooked ham from one manufacturer. With all previous outbreak reports in mind, this outbreak emphasises, once again, the risk for infection with *L. monocytogenes* from deli meats. Therefore, we advocate for updating dietary and hygiene recommendations for persons at risk of listeriosis. Packaged sliced sausages and other cold cuts, even if parboiled or cooked, should be treated and clearly communicated as high-risk foods. In healthcare settings, these foods should either not be consumed at all or be served freshly sliced to avoid the multiplication of *L. monocytogenes* after a potential contamination during the slicing and packaging process.

## Supplementary Data

1039000 Supplementary Material
