# Supplementary material for: Outbreak of listeriosis associated with consumption of deli meats in a hospital, Germany, February to March 2023
Source: Euro Surveill. 2025 Feb 20;30(7):2400316. doi: 10.2807/1560-7917.ES.2025.30.7.2400316 (PMC11843619; doi:10.2807/1560-7917.ES.2025.30.7.2400316)
Supplement: Supplementary Material [file 24-00316_SCHOEPS_Supplement.pdf]

This supplementary material is hosted by Eurosurveillance as supporting information alongside the article "Nosocomial listeriosis outbreak: time to rethink supply, preparation, and consumption of sausages and other deli meats in the health care setting in Germany", on behalf of the authors, who remain responsible for the accuracy and appropriateness of the content. The same standards for ethics, copyright, attributions and permissions as for the article apply. Supplements are not edited by Eurosurveillance and the journal is not responsible for the maintenance of any links or email addresses provided therein.

Supplementary Table 1: Details of environmental and food samples

Sample information					Laboratory result	
**Type of sample**	**Expiry date**	**Batch No**	**Collection date**	**Collection place**	**Date**	**Detection of microbiological pathogen**
**Packaged mixed sliced sausages, breakfast (food_1)**	-	1	14.03.2023	hospital A	23.03.2023	*L. monocytogenes*
**Packaged mixed sliced sausages, dinner (food_2)**	-	1	14.03.2023	hospital A	23.03.2023	*L. monocytogenes*
**Whole scalded turkey sausage (*Putenwiener*)**	-	-	14.03.2023	hospital A	20.03.2023	none
**Whole cooked turkey ham (*Putenkochschinken*)**	-	-	14.03.2023	hospital A	20.03.2023	none
Whole scalded pork sausage (***Fleischwurst***)	-	-	14.03.2023	hospital A	20.03.2023	none
Surface sample left work table	-	-	23.03.2023	meat supplier A	03.04.2023	*L. welshimeri*
Surface sample slicer, left knife (slicing and packaging room)	-	-	23.03.2023	meat supplier A	03.04.2023	none
Surface sample slicer, right knife (slicing and packaging room)	-	-	23.03.2023	meat supplier A	03.04.2023	none
Surface sample aluminum working table	-	-	23.03.2023	meat supplier A	03.04.2023	none
Surface sample slicer, left knife (slicing and packaging room)	-	-	23.03.2023	meat supplier A	03.04.2023	none
Surface sample slicer, right knife (slicing and packaging room)	-	-	23.03.2023	meat supplier A	03.04.2023	none
Surface sample sink (slicing and packaging room, env_1)	-	-	23.03.2023	meat supplier A	03.04.2023	*L. monocytogenes*
Surface sample slicer, left knife (slicing and packaging room)	-	-	23.03.2023	meat supplier A	03.04.2023	none
Surface sample slicer, right knife (slicing and packaging room)	-	-	23.03.2023	meat supplier A	03.04.2023	none
Surface sample aluminum working table, white chopping board (slicing and packaging room)	-	-	23.03.2023	meat supplier A	03.04.2023	none
Surface sample sewer (slicing and packaging room)	-	-	23.03.2023	meat supplier A	03.04.2023	none
Surface sample sewer (slicing and packaging room, env_2)	-	-	23.03.2023	meat supplier A	03.04.2023	*L. monocytogenes*, *L. welshimeri*
Surface sample hand wash basin	-	-	23.03.2023	meat supplier A	03.04.2023	none
Surface sample working table (new packaging room)	-	-	23.03.2023	meat supplier A	03.04.2023	none
Surface sample scale (new packaging room)	-	-	23.03.2023	meat supplier A	03.04.2023	none
Surface sample slicer, left knife (new packaging room)	-	-	23.03.2023	meat supplier A	03.04.2023	none
Surface sample slicer, right knife (new packaging room)	-	-	23.03.2023	meat supplier A	03.04.2023	none
Surface sample slicer, belt chains (new packaging room)	-	-	23.03.2023	meat supplier A	03.04.2023	none
**Whole scalded turkey sausage (Putenlyoner)**	-	-	23.03.2023	meat supplier A	03.04.2023	none
Whole pork ham (***Formfleischhinterschinken***)	-	-	23.03.2023	meat supplier A	03.04.2023	none
Packaged sliced scalded pork sausage, 800gr (***Bierwurst***)	-	-	24.03.2023	hospital A	28.03.2023	none
Packaged sliced scalded beef and pork sausage, 500gr (***Bierwurst***, food_3)	28.03.2023	2	24.03.2023	hospital A	28.03.2023	*L. monocytogenes*

Packaged sliced cooked turkey ham (***Putenkochschinken***)	-	-	24.03.2023	hospital A	28.03.2023	none
Packaged sliced cooked turkey ham (***Putenkochschinken***)	-	-	24.03.2023	hospital A	28.03.2023	none
**Packaged whole scalded pork sausages (*Wiener Würstchen*)**	-	-	24.03.2023	hospital A	28.04.2023	none
**Packaged whole scalded scalded pork sausages (*Wiener Würstchen*)**	-	-	24.03.2023	hospital A	28.04.2023	none
Packaged sliced cooked pork ham (***Hinterkochschinken***, food_4)	26.03.2023	3	29.03.2023	caterer A	11.04.2023	*L. monocytogenes*
**Whole scalded pork sausage (*Pfefferlyoner*)**	-	-	03.04.2023	meat supplier A	-	none
Whole scalded pork sausage (***Bierschinken***)	-	-	03.04.2023	meat supplier A	-	none
Whole scalded pork sausage (***Schwartenmagen***)	-	-	03.04.2023	meat supplier A	-	none
**Whole scalded pork sausage (*Champignonlyoner*)**	-	-	03.04.2023	meat supplier A	-	none
**Whole scalded pork sausage (*Jagdwurst*)**	-	-	03.04.2023	meat supplier A	-	none
**Whole scalded pork sausage (*Paprikalyoner*)**	-	-	03.04.2023	meat supplier A	-	none
**Whole scalded pork sausage (*Lyoner*)**	-	-	03.04.2023	meat supplier A	-	none
Surface sample aluminum work table	-	-	03.04.2023	meat supplier A	-	none
Surface sample sewer 1	-	-	03.04.2023	meat supplier A	-	none
Surface sample slicer 1	-	-	03.04.2023	meat supplier A	-	none
Surface sample white working table	-	-	03.04.2023	meat supplier A	-	none
Surface sample slicer	-	-	03.04.2023	meat supplier A	-	none
Surface sample slicer 2	-	-	03.04.2023	meat supplier A	-	none
Surface sample filling machine	-	-	03.04.2023	meat supplier A	-	none
Surface sample cutting board 1 (disassembly room)	-	-	03.04.2023	meat supplier A	-	none
Surface sample cutting board 2 (disassembly room)	-	-	03.04.2023	meat supplier A	-	none
Surface sample wall (cold room)	-	-	03.04.2023	meat supplier A	-	none
Surface sample sewer 2	-	-	03.04.2023	meat supplier A	-	none

* below legally established European threshold of 100 CFU/gram
Names in italics represent original designation of food items in German language

Supplementary Table 2: Characteristics of the outbreak strain food_1

**isolate ID**	**food_1**
**outbreak cluster**	Lambda14
**source type**	food isolate
**source of isolation**	deli meat (scalded sausage)
**year of isolation**	2023
**genome size**	3 Mbp
**serogroup**	IVb
**MLST sequence type**	2
**cgMLST complex type**	2834
**MLST clonal complex**	CC2
**resistance genes (ResFinder)**	fosfomycin (*lmo1702*|CAC99780.1)
**pathogenicity islands**	LIPI-1
**virulence genes (VirFinder)**	*AgrA, FlaA, FlgC, FlgE, GadB, GadC, Gmar, Lap, LapB, OatA, OppA, OrfX, OrfZ, Rli55, Rli60, Rsbv, biLE, bsh, btlB, chiA, clpB, clpc, clpe, clpp, codY, ctaP, ctsR, dal, degU, dltA, fbpA, fri, fur, hfq, hly, htrA, hupC, iap, inlA, inlB, inlC, inlH, inlJ, lgt, lhrC, lhrC, lhrC, lipA, lisR, lmo0514, lmo2085, lntA, lpeA, lplA1, lsp, mogR, mpl, mprf, murA, perR, pgdA, pgl, plcA, plcB, prfA, prsA2, pycA, recA, relA, secA2, sigB, sipX, sipZ, sod, srtA, srtB, stp, svpA, tcsA, tig, uHpt, virR*

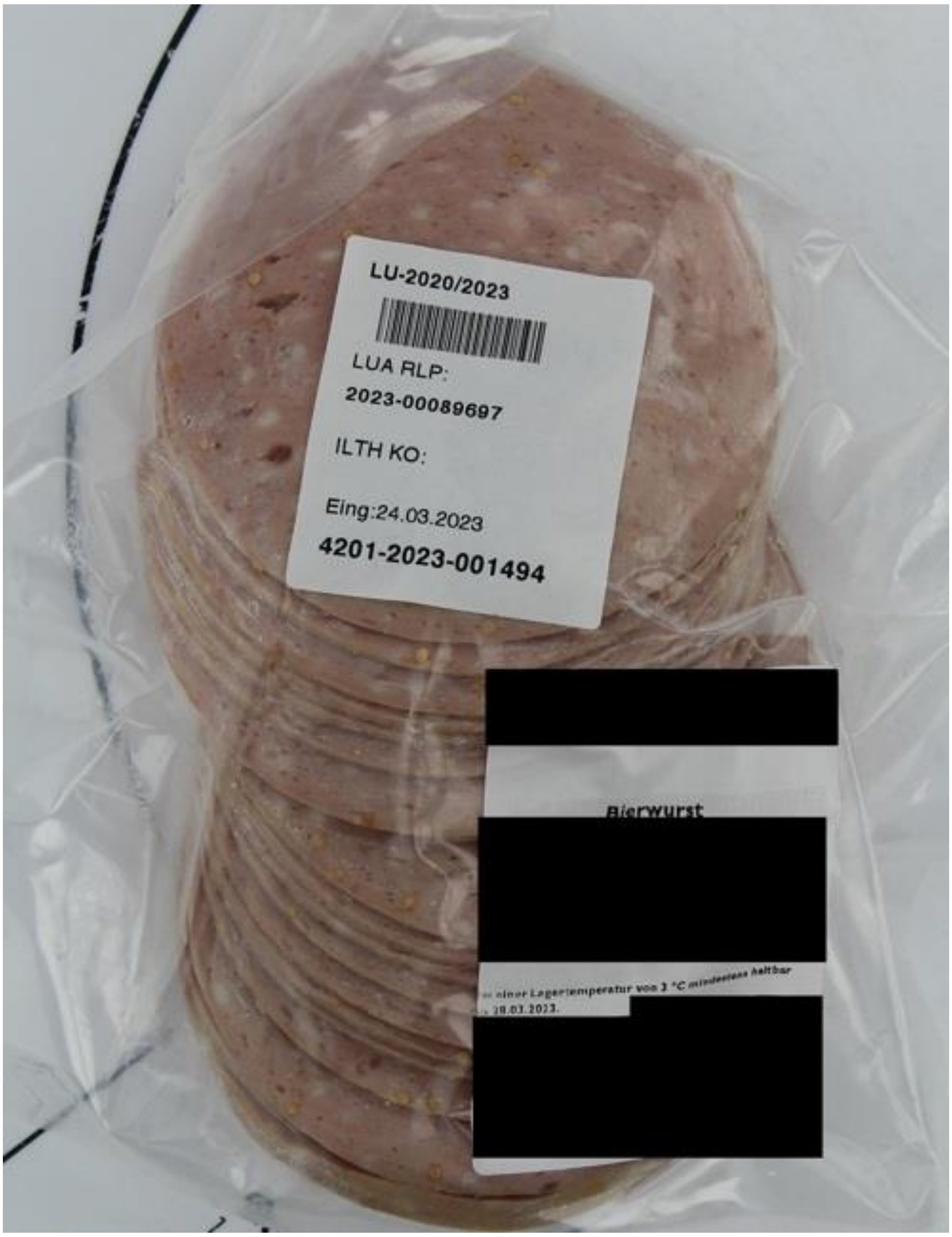

Supplementary Figure 1: Photo of the packaged sliced scalded beef and pork sausage, 500gr (*Bierwurst*, food_3), which was analysed at the federal state laboratory of Rhineland-Palatinate, Germany. *L. monocytogenes* was detected in a sample of 25 grams but contamination was below the legally acceptable threshold of 100 CFU per gram of the product.

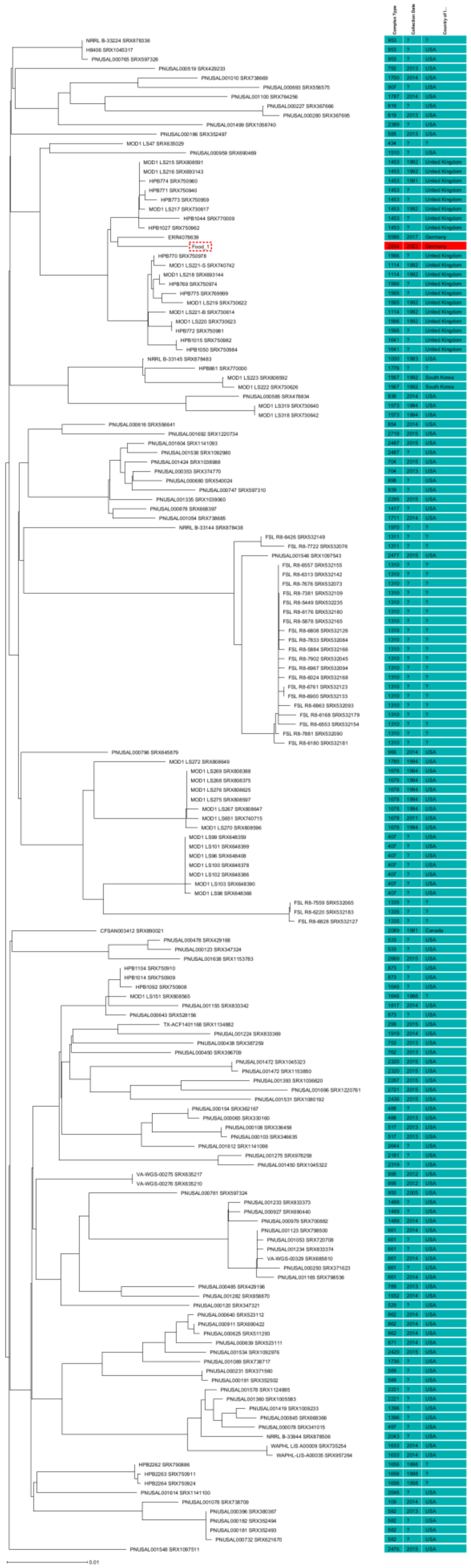

Supplementary Figure 2: Neighbour-joining tree based on cgMLST data comparing 1385 genes of 162 publicly available sequences of *L.monocytogenes* MLST ST2 isolates including the outbreak strain food_1 [28]. The outbreak strain food_1 (coloured in red) has 22 allele and 79 SNP differences to a clinical *L. monocytogenes* isolate submitted in 2017 by the Robert Koch Institute (Biosample: SAMEA6800831).
